# Medical optics in medical physics: from photons to quantitative insight

**DOI:** 10.1016/j.zemedi.2026.03.006

**Published:** 2026-03-14

**Authors:** Achim Langenbucher, Sibylle Scholtz

**Affiliations:** Department of Experimental Ophthalmology, Saarland University, Homburg/Saar, Germany

Medical optics is among the most rapidly advancing domains within medical physics, owing to the unique ability of light to probe tissue non-invasively with high spatial and temporal resolution and a wide range of contrast mechanisms. Recent progress has been driven not only by improved optical components but also by the system-level integration of optical design, computational reconstruction, automated analysis, and physics-based modelling. This Special Issue illustrates how such integration translates into more patient-friendly measurements, more robust quantification, and clearer links between optical metrics and clinical decision-making.

Medical-optical methods rest on established physical principles—refraction, interference, scattering, absorption, and coherence—yet recent years have seen a pronounced shift from image acquisition alone toward **deployable quantification**: measurement pipelines that are reliable, interpretable, and efficient in clinical workflows. Ophthalmology has served as a particularly fertile testbed for this evolution, as the eye is optically accessible while simultaneously demanding high precision. The forward-looking review on **in vivo corneal microscopy** synthesizes where in vivo confocal microscopy (IVCM) stands today and where it is heading. It highlights innovations aimed at extending the field of view, reducing operator dependence through automation, and improving patient comfort so that cellular-resolution information can support broader clinical adoption [7].

Two original contributions translate these goals into concrete technical advances. The non-contact IVCM approach for corneal endothelium imaging demonstrates that cellular-resolution assessment can be achieved without corneal contact or topical anesthesia, and—importantly—can be extended from the central cornea toward near-limbal regions. This broadened sampling is clinically relevant, as peripheral endothelial characteristics may differ from central measures and are often difficult to capture with standard techniques [8].

Complementing this work, the low-cost optical add-on that converts a retinal OCT system into an ultra-high-resolution corneal OCT exemplifies a pragmatic route to innovation: leveraging a widely available platform while implementing targeted optical modifications to deliver substantial gains in lateral resolution. The resulting improvement in the visibility of fine epithelial microstructures and subtle corneal irregularities illustrates how carefully engineered upgrades—rather than complete platform replacement—can expand diagnostic capability while lowering barriers to adoption and training [9].

High-quality measurement, however, represents only one side of precision; the other is **modelling**. As optical imaging provides increasingly detailed geometric information, physics-based simulation becomes indispensable for translating measured anatomy into optical predictions. The ray-tracing study on the **impact of the corneal epithelium** addresses this challenge directly, by using OCT-derived point-cloud representations and multi-surface corneal models to investigate how epithelial structure influences corneal power estimation. Beyond its clinical motivation, the work underscores a broader methodological point relevant to medical physics: robust surface representation and careful handling of measurement noise are prerequisites for stable, interpretable simulation outcomes—particularly in irregular corneas and post-surgical geometries [3].

A second raytracing contribution advances model-based optics from evaluation to design. By applying optimization to derive **best shape spherical and aspherical intraocular lens geometries** that minimize wavefront error and reduce spherical aberration, the study aligns with a wider trend in ophthalmic optics: moving from standardized optical components towards performance-driven, model-informed designs assessed using physically meaningful metrics [4].

Together, these modelling papers highlight an emerging standard in the field: higher measurement resolution must be matched by validation-grade modelling, well-defined endpoints, and transparent conventions to ensure that “more data” truly becomes “better prediction”.

Medical optics also depends critically on the mathematics of clinical measurement. The analysis of coordinate systems in the **Hess screen test** and the **Harms tangent screen test** provides a rigorous derivation of their metrics and formal relationships, demonstrating mathematical equivalence while emphasizing that numerical values and interpretation depend on the underlying angle definitions and test procedures. Such clarity is essential for meaningful cross-method comparison, for avoiding systematic interpretive errors, and for supporting reliable digital implementations of diagnostic workflows [6].

Beyond imaging and geometry, this issue reflects the expanding role of light as an actuator in biomedical systems. The study on **intensity modulation of frequency-specific optoacoustic stimulation** at the tympanic membrane addresses a foundational requirement for translation in optical stimulation paradigms: controlled mapping between optical drive parameters, mechanical response, and physiological output. By linking ex vivo mechanical characterization to in vivo neural responses, the work strengthens the physical basis for developing intensity-graded, frequency-specific optoacoustic strategies relevant to future auditory prosthesis concepts [1].

A further dimension of medical optics is the shift from structural imaging to objective functional assessment, addressing the long-standing challenge of **quantifying accommodation after implantation of an accommodating intraocular lens**. Methodology-focused contributions of this kind are essential, as subjective near performance may be confounded by depth of focus, pupil dynamics, and neuroadaptation. In contrast, objective protocols anchored in reproducible optical endpoints are needed to enable comparability across devices, lens concepts, and patient cohorts, and to guide future design iterations toward measurable and clinically meaningful performance [2].

Finally, innovation in medical optics does not occur in isolation; it depends on education, communication, and a vibrant scientific culture. The short communication on the **Deutsches Optisches Museum** (German Optical Museum) **in Jena** presents a modern “edutainment” concept for a newly established national museum scheduled to open in 2028. It bridges optics and photonics with medical physics through interactive exhibits, historical instruments, and public engagement, including themes relevant to ophthalmic optics. Such initiatives foster literacy in measurement principles, strengthen trust in technology, and inspire future contributions to a field whose most impactful advances often become “invisible” once embedded in routine clinical devices [5].

## Outlook

Taken together, the contributions in this Special Issue demonstrate how medical optics is evolving towards **patient-friendly implementation, workflow-compatible innovation, and model-based quantification**, underpinned by mathematical clarity and cross-domain validation. We hope this collection stimulates further collaboration across optical engineering, computational methods, and clinical translation, thereby accelerating the path from physical principle to measurable patient benefit.

Across all contributions, several trajectories emerge clearly: (1) **resolution and coverage** are being improved not only through new devices, but also through clever re-engineering of existing platforms; (2) **non-contact and patient-friendly approaches** are gaining momentum; (3) **model-based optics** — raytracing, surface representation, optimization — is moving closer to clinical workflows; and (4) rigorous **definitions and transformations** remain indispensable for reliable interpretation. The coming years will likely deepen this convergence of optics, computation, and clinical translation. Medical optics is entering a phase in which the most decisive innovations are those that make precision clinically *deployable*: robust, affordable, comfortable — and quantitatively interpretable.

## References

[1] Dries P., Lim H.H., Hinsberger M., Sorg K., Pillong L., Langenbucher A., Schick B., Wenzel G.I. (2026). Intensity modulation of frequency-specific optoacoustic stimulation at the peripheral hearing level. Z Med Phys.

[2] Eppig T., Müller V., Seer M., Bölke A., Meßner A., Rombach M., Becker E., Schrecker J. (2026). Objective assessment of accommodation after implantation of an accommodating intraocular lens: methodology and case report. Z Med Phys.

[3] Langenbucher A., Szentmáry N., Cayless A., Hoffmann P., Wendelstein J. (2026). Impact of the corneal epithelium on the corneal power using 3D raytracing with OCT data. Z Med Phys.

[4] Langenbucher A., Wendelstein J., Cayless A., Hoffmann P., Szentmáry N. (2026). Deciphering the best shape aspheric intraocular lens - A raytracing based optimisation study. Z Med Phys.

[5] Mappes T. (2026). Deutsches Optisches Museum - edutainment for optics & photonics. Z Med Phys.

[6] Oltrup T., Bende M., Bende T., Leitritz M.A., Bartz-Schmidt K.U. (2026). The coordinate systems in the Hess screen test and the Harms tangent screen test for ocular motility analysis of strabismus: their metrics and relationship. Z Med Phys.

[7] Sperlich K., Bohn S., Worsch F., Colorado L.H., Allgeier S., Stachs O. (2026). In vivo corneal microscopy: from image to insight - where are we going?. Z Med Phys.

[8] Sperlich K., Gottschlich A., Winter K., Worsch F., Stachs O., Bohn S. (2026). Design, implementation and evaluation of non-contact in vivo confocal microscopy for capturing the human corneal endothelium from the central to near-limbal regions. Z Med Phys.

[9] Stange F., Bohn S., Gottschlich A., Korn T., Stachs O., Sperlich K. (2026). A low-cost, easy-to-implement optical add-on to convert a retinal OCT into a ultra-high-resolution corneal OCT. Z Med Phys.

